# Oxidative and Antioxidative Stress Status in Children with Inflammatory Bowel Disease as a Result of a Chronic Inflammatory Process

**DOI:** 10.1155/2018/4120973

**Published:** 2018-06-11

**Authors:** Urszula Grzybowska-Chlebowczyk, Paulina Wysocka-Wojakiewicz, Martyna Jasielska, Bożena Cukrowska, Sabina Więcek, Maria Kniażewska, Jerzy Chudek

**Affiliations:** ^1^Department of Pediatrics, School of Medicine in Katowice, Medical University of Silesia, Katowice, Poland; ^2^Upper Silesian Child Health Center, Katowice, Poland; ^3^Department of Pathology, The Children's Memorial Health Institute, Warsaw, Poland; ^4^Department of Pathophysiology, School of Medicine in Katowice, Medical University of Silesia, Katowice, Poland

## Abstract

Oxidative stress (OS) has been recently implicated in the disease pathogenesis in inflammatory bowel disease (IBD). The aim of the study was to evaluate oxidative and antioxidative stress status and the risk of the atherosclerotic process in children with IBD and functional gastrointestinal disorders (FGID). The prospective study included a group of 71 children during a period of 2 years. In all children, laboratory tests were performed and intima-media complex in the carotid artery was measured (IMC). Low values of OS were more frequent in children with IBD than in the FGID group. The average concentration of oxidized lipoprotein with average density (oxLDL) was lower in patients with IBD. Among patients with IBD, higher concentrations of oxLDL were recorded in patients with longer-duration disease and with higher concentrations of total cholesterol. In the IBD group, more often, higher concentrations of anti-oxLDL were recorded among patients with longer-duration disease. The obtained results did not support the hypothesis of total antioxidant capacity depletion and greater overall OS in patients with IBD. Patients with IBD with a longer duration of the disease have higher concentrations of oxLDL and anti-oxLDL.

## 1. Introduction

Inflammatory bowel disease (IBD), ulcerative colitis, and Crohn's disease are chronic gastrointestinal diseases with unclear etiology. Their clinical presentation is dominated by chronic inflammatory process affecting the gastrointestinal tract and with extraintestinal manifestation in 25–35% of patients. The etiopathogenesis of these diseases is multifactorial. Several environmental, immunological, microbiological, and genetic factors are involved in the pathogenesis of IBD [1]. Recently, oxidative stress also has been implicated in the disease pathogenesis and/or development [2].

In healthy people, the action of reactive oxygen species (ROS) is counteracted by antioxidants, substances causing the conversion of superoxide radicals into inactive derivatives and constitute the so-called antioxidant protection system. Insufficient antioxidant protection or excessive production of ROS generates a condition known as oxidative stress. The production of ROS associated with chronic inflammation contributes to the impaired function of the vascular endothelium. The term “endothelial dysfunction” refers to reduced production or availability of the main relaxing factor—nitric oxide (NO)—and increase in the shrinking factors, including endothelin-1, angiotensins, and oxidants, which lead to the stenosis of blood vessels [3]. This endothelial dysfunction of blood vessels is regarded as one of the etiological factors in inflammatory bowel diseases (IBD) [4].

Changes in the microcirculation of the bowel, especially impaired vasodilatation in IBD, may lead to intestinal hypoperfusion with a subsequent damage. It has been shown that increased ROS in chronic inflammation with excessive levels of oxidative stress contributes to endothelial dysfunction followed by dysregulation of intestinal blood supply and impaired healing of ulcers [5].

As a result of the overproduction of reactive forms of oxygen exceeding the physiological capacity of the antioxidant systems, the process of lipid peroxidation enhances. Oxidized low-density lipoproteins are created in this way—oxLDL—which take part in stimulating the processes of atherogenesis [6–8]. The production of free radicals and changes in which they are involved lead to tissue remodeling and the intensification of the process of atherosclerosis. It has been proven that in patients with IBD, functional changes in blood vessels appear before structural changes [9]. Currently, we can assess in a noninvasive way the presence and severity of the process of atherosclerosis, thanks to the Doppler evaluation of IMC (intima-media complex) of common carotid arteries. It has been shown that the value of IMC in carotid arteries correlates with the severity of the atherosclerotic process in other arterial vessels [10]. The SMART survey showed that in adults, the measurements of IMC can be regarded as prognostic factors for the risk of vascular incidents [11]. In addition, we know that the cardiovascular risk (measured as the frequency of heart attacks and strokes) increases with the thickness of IMC [12–14]. Research on IMC in children and youth pointed its predictive role in the detection of cardiovascular risk factors [15, 16].

There are several gastrointestinal diseases such as peptic ulcer, *Helicobacter pylori* infection, and gastroparesis associated with antioxidant dysfunction. In addition, psychological stress was shown to accelerate oxidative stress in functional gastrointestinal disorders (FGID) [17].

Nowadays, we can assess an overall oxidation status (total oxidant status (TOS)) [18] and antioxidant status of the body (total antioxidant status (TAS)) [19] and calculate the TOS-to-TAS ratio called oxidative stress index (OSI) [20]. OSI is considered a more precise measure of oxidative stress status in the body [21].

There are a limited number of studies especially in children investigating oxidative stress and antioxidative protection system status in IBD and FGID. Therefore, the aim of the study was to evaluate oxidative and antioxidative stress status and the risk of atherosclerotic process in children with IBD and functional gastrointestinal disorders.

## 2. Material and Method

The prospective study included a group of 71 children diagnosed and treated for IBD in the Department of Gastroenterology, Department of Pediatrics, Medical University of Silesia, in Katowice during a period of 2 years. There were 47 boys and 24 girls, with ages ranging from 3 to 18 years. Crohn's disease occurred in 35 children (49.3%), ulcerative colitis in 36 children (50.7%). The characteristics of the study group are shown in Table 1.

The control group consisted of 29 children (21 girls and 8 boys) diagnosed with FGID based on Rome III criteria, with normal anthropometric parameters, normal blood pressure, and normal values in the basic lab tests.

In children in both groups, anthropometric measurements (body weight, height) were performed, *Z*-score BMI was calculated, and peripheral blood samples were taken for lipidogram, total antioxidative status/capacity (TAS/TAC), total oxidative status/capacity (TOS/TOC), oxLDL, anti-oxLDL, and antibodies ANCA and ASCA; also, intima-media complex in the carotid artery was measured (IMC).

Total antioxidative status/capacity (TAS/TAC) was determined photometrically by an enzymatic reaction using a commercially available kit provided by Immundiagnostik AG (Bensheim, Germany). Intra-assay variability < 4%. Values < 280, 280–320, and >320 *μ*mol/l were scored as low-, middle-, and high-antioxidative capacity, respectively. Total oxidative status/capacity (TOS/TOC) was determined by the reaction of peroxidase with peroxides using commercially available kit provided by Immundiagnostik AG (Bensheim, Germany). Intra-assay variability < 3%. Values < 200, 200–350, and >350 *μ*mol/l were scored as low-, moderate-, and high-oxidative stress, respectively. The TOS-to-TAS ratio was regarded as the oxidative stress index (OSI) [18]. oxLDL (MDA-modified Apolipoprotein B 100, containing less than 60 MDA units per molecule) were measured by ELISA using commercially available kit provided by Immundiagnostik AG (Bensheim, Germany). Intra-assay variability < 6%. Anti-oxLDL antibodies were measured by ELISA using a commercially available kit provided by Immundiagnostik AG (Bensheim, Germany). Intra-assay variability < 7%. Antineutrophil cytoplasm antibodies (ANCA) in immunoglobulin class G (IgG) and IgA and IgG anti-*Saccharomyces cerevisiae* antibodies from ANCA and ASCA were determined using commercial Euroimmun ELISA tests.

Additionally, in 70 children (50 patients from the test group and 20 from the control group), a measurement of the thickness of the intima-media of the carotid artery was performed using an ultrasound method—a linear probe with a frequency of 1–15 MHz in M-mode view with the ultrasound machine Aloka prosound SSD 3500sx on 3 locations of the common carotid artery. The result representing the intima-media complex (IMC) is presented as an average of the measurements taken.

Diagnosis of the disease in the study group was based on revised Porto [22] criteria—clinical data and macroscopic and microscopic images of the bowel in endoscopic examination as well as magnetic resonance imaging. Disease progression was assessed using *Pediatric Ulcerative Colitis Activity Index* (PUCAI) and *Pediatric Crohn's Disease Activity Index* (PCDAI) [23, 24].

The research was approved by the Bioethics Committee of the Silesian Medical University, approval number KNW/0022/KB1/131/I/14 and written consent was obtained from participants and/or their parents, as appropriate.

Statistical analysis was performed based on the procedures available in the licensed software MedCalc 14.8.1 Ostend, Belgium. Quantitative variables due to not-normal distribution, verified by Kolmogorov–Smirnov test, were presented as medians and interquartile range. Qualitative variables were expressed in terms of absolute value and percentage. Intergroup differences for quantitative variables were evaluated by the Mann–Whitney *U* or Kruskal–Wallis test. For qualitative variables, the chi-square test or Fisher's exact test was used. Correlations were interpreted based on Spearman's correlation coefficient analysis. The results of simple analysis were verified using multivariable analysis in a multiple regression model. Models included variables for which *p* < 0.1 in simple analysis. The criterion of statistical significance was set at *p* < 0.05.

## 3. Results

The prevalence of high levels of total oxidative stress (TOS/TOC) was similar in patients with Crohn's disease (77%), ulcerative colitis (72%), and FGID (82%). Low values of oxidative stress were more frequent in children with UC (*N* = 8; 22.2%) and CD (*N* = 5; 14.3%), than in the FGID group (*N* = 2; 6.9%). However, these differences were not statistically significant (*p* = 0.9, Table 2).

Also, the distribution of total antioxidative status (TAS/TAC) was not significantly different among the groups. In the majority of patients, both in the CD (*N* = 22; 62.8%) and UC (*N* = 23; 63.8%) and in FGID (*N* = 23; 79.3%) groups, TAS/TAC was low (below the value of <280 *μ*mol/l). Furthermore, the values of oxidative stress index (OSI) was similar in all 3 groups (Table 2).

Markers, TOS/TOC, TAS/TAC, and OSI, were not associated with the duration and activity of IBD, occurrence of ANCA, body mass index, and BMI *Z*-score (Table 3).

The average concentration of oxidized lipoprotein with average density (oxLDL) was lower in patients with inflammatory bowel diseases, with Crohn's disease (213.9 ng/ml), and with ulcerative colitis (141.3 ng/ml) than in children with FGID (277.6 ng/ml) (Table 2). Among patients with IBD, statistically significantly higher concentrations of oxLDL were recorded in patients with longer-duration disease (*p* = 0.01) and with higher concentrations of total cholesterol and HDL (*p* < 0.05). In children with remission of the underlying disease, the concentration of oxLDL was higher (*p* = 0.04); however, it should be mentioned that among these children, 42.8% were in the course of immunosuppressive treatment and among children with an active disease, as much as 70% (Table 3). There was no relationship between the use of immunosuppressive drugs and concentration of oxLDL (*p* > 0.05). Patients with FGID had the highest concentration of total cholesterol and LDL cholesterol, and this difference was statistically significant (*p* < 0.05). Post-hoc comparisons were performed. It was revealed that the total cholesterol level was significantly higher in FGID compared to both the CD and CU groups, with no difference between CD and CU subjects. Similar findings were found for LDL concentration.

The highest concentration of anti-oxLDL was found in a group of children with CD (on average 8058 U/ml), and the lowest in children with FGID; however, this difference was not statistically significant (*p* > 0.05). In the group of children with IBD, more often, statistically significantly higher concentrations of anti-oxLDL were recorded among patients with longer-duration disease (*p* = 0.03). The existence of a positive correlation was found between the concentration of oxLDL and that of anti-oxLDL (*p* < 0.001).

Similar thickness of the carotid artery intima-media complex (IMC) was found in children with ulcerative colitis Crohn's disease and FGID, and the obtained results remained within the normal range for the age of patients.

ANCA, ASCA IgA, and ASCA IgG antibodies were tested in 33 patients with Crohn's disease, in 32 with ulcerative colitis, and in 21 with FGID. ASCA IgA were detected in 18 patients (54.5%) with Crohn's disease, what has been statistically significant more often than in patients with FGID (14.2%) and UC (none) (*p* < 0.001). Similarly, ASCA IgG have been statistically significantly more likely (*p* < 0.001) positive in children with Crohn's disease (54.2%) than FGID (4.7%) and ulcerative colitis (none). In addition, two children with Crohn's disease (8%) and one with ulcerative colitis (4%) were ANCA positive (*p* = 0.4).

ASCA IgA-positive children with Crohn's disease had lower TAS and TOS than the seronegative CD subgroup (*p* = 0.06). However, ASCA IgG-positive children with Crohn's disease had lower oxLDL and anti-oxLDL than the seronegative CD subgroup (*p* < 0.05).

## 4. Discussion

The obtained results showed higher concentrations of oxLDL and anti-oxLDL among children with IBD with longer duration of the disease and higher concentrations of total cholesterol and HDL. Total antioxidant capacity in children with IBD and FGID was similar, and there was no correlation between IBD severity and all analyzed markers of the oxidative status (TOS/TOC, TAS/TAC, and OSI). Only ASCA IgA-positive children with Crohn's disease had slightly and almost significantly lower TAS/TOS than the seronegative subgroup. However, in patients with CD and a positive result of ASCA IgG, oxLDL and anti-oxLDL were lower than in seronegative patients.

Patients with nonspecific inflammatory bowel diseases are at greater risk of cardiovascular disease, including atherosclerosis, for many reasons. Oxidative stress, endothelial dysfunction, stiffness of blood vessels, and disturbances of the intestinal microbiota are taken into account. Kocaman et al. proved that both dependent and independent endothelial dilation of blood vessels depend on the illness severity and are more severe in patients with a severe or moderate form of colitis ulcerosa and the endothelial dysfunction is associated with parenteral complications dependent of the intestinal disease activity [4]. In previous studies in patients with IBD, an increased stiffness of blood vessels was observed [4, 25], additionally increasing the risk of cardiovascular disease. The stiffness of blood vessels can be reduced by the use of immunomodulating therapy with the use of steroids and azathioprine or anti-TNF-*α* drugs. It is believed that the effective long-term control of inflammation can reduce the cardiovascular risk in patients with IBD, by affecting the stiffness of blood vessels [26, 27]. However, in our study, no relationship was found between the use of the immunosuppressive treatment and the concentration of oxLDL in patients with IBD; however, this may be related to the fact that the study was conducted in children, in which the duration of the disease and the length of the treatment were relatively brief (average duration of the disease 28 months).

Our patients with inflammatory bowel diseases, and in particular patients with Crohn's disease, had reduced concentration of both total and LDL cholesterol. Hrabovský et al. demonstrated that altered metabolism of lipids is a feature of the active disease and CD patients in the disease flare have decreased cholesterol levels among other abnormalities [28]. Furthermore, our examined patients with IBD had a lower concentration of oxLDL than patients with functional gastrointestinal disorders. However, it should be noted that patients with longer duration of inflammatory bowel disease were statistically significantly more likely to have a higher concentration of oxLDL than patients with shorter duration of the disease, which is consistent with Boehm and colleague's reports in which adult patients with Crohn's disease, especially in the active phase, have lower concentrations of oxLDL and peroxidation potential and a high level of oxLDL was positively correlated with the level of total cholesterol [29]. Additionally, previous studies have shown that under the influence of oxLDL T lymphocytes, cellular response and production of anti-oxLDL antibodies are activated. This is confirmed by the already raised thesis about autoaggressive stimulation of the immune system by oxLDL [15, 16]. The highest concentration of anti-oxLDL in our study was found in children with Crohn's disease, and in addition, statistically significantly higher concentrations were recorded in patients with longer disease duration. Previous studies of the intima-media complex in the carotid artery in children and youth indicated its predictive role in the detection of cardiovascular risk factors [25, 30]. Therefore, it was suggested that the measurement of IMC can be used to identify people with high risk for cardiovascular events among patients with IBD. However, the research of Broide et al. and Üstün et al. has shown that the values of IMC in patients with IBD are similar to the values of healthy subjects and patients with IBD are exposed to an increased risk of accelerated atherosclerosis [15, 16]. In our study, the largest thickness of the intima-media complex has been shown in patients with ulcerative colitis, and the smallest in children with Crohn's disease; however, this difference was not statistically significant, and the obtained results among children patients and healthy subjects were within normal limits for the age of examined patients. Similarly to the test results of IMC, the test results of the lipidogram were normal; therefore, we evaluated the cardiovascular risk of examined children as low.

IBD causes significant changes in neurally controlled gut functions. Clinical symptoms are caused at least in part by prolonged hyperexcitability of enteric neurons that can occur in the course of colitis. The immune cells like enterochromaffin cells and mast cells are increased in the colonic mucosa, including 5-hydroxytryptamine and cytokines, as well as ROS [31]. Aslan et al. showed significantly higher values of TOS and OSI but lower TAS in patients with UC compared to the control group. TAS level correlated with those of TOS and OSI [26]. Also, a recently published study showed a two-fold increase in TOS in patients with IBD, unrelated to the type of disease, compared to controls [32]. It is worth mentioning that in most studied patients with IBD, concentrations of TOS and TAS were not dependent on the severity, location, or type of disease as well as the therapy [33]. Also, in our study, the levels of TOS and TAS in children with IBD were not correlated with the type of disease and disease activity. Only Kontroubakis showed higher levels of TAS in patients with proctitis in UC compared to patients with involvement of the left and the entire colon. Inflammation causes disturbances of intestinal barrier function, abnormal secretion, motility changes, and visceral sensation, which contributes to symptom generation. Therefore, it seems that the effect of local generation of ROS on antioxidative status of the whole body is limited.

We utilize the group of children with FGID as controls, as we were able to exclude even subclinical bowel inflammation based on detailed differential diagnosis. TOS and TAS studies have not been performed in children with functional gastrointestinal disorders before. Probably in this group of patients, we can also look for the cause of clinical symptoms in the markers of oxidative stress. In our study, the levels of TOS, TAS, and OSI were similar in children with IBD and FGID. FGID has been considered as a condition resulting from brain-gut dysregulation, and the symptoms are not explained by structural or biochemical abnormalities. However, even in such patients, we cannot rule out the impact of oxidative stress on the pathogenesis of intestinal dysmotility that may be related to altered mucosal and immune function, and gut microbiota and low-grade activation of mast cells, and increased inflammatory cytokine release [34]. Mete et al. showed high TOS and low TAS in patients with irritable bowel syndrome compared to controls [35]. We also obtained similar results, which may indicate the important role of oxidative stress in the pathogenesis of functional gastrointestinal disorders. The limitation of our study was the lack of a group of healthy children in order to compare, and the limited number of observations might weaken the power of the analysis. Also due to the shorter duration of the disease than in adults, it is difficult to assess the impact of stress on the development of atherosclerotic processes that enhance along with age; so, further prospective study should be preformed.

## 5. Summary

The obtained results did not support the hypothesis of total antioxidant capacity depletion and greater overall oxidative stress in patients with inflammatory bowel disease. Patients with IBD with a longer duration of the disease have higher concentrations of oxLDL and anti-oxLDL. However, due to the shorter duration of the disease than in adults, it is difficult to assess the impact of stress on the development of atherosclerotic processes that enhance along with age.

## Figures and Tables

**Table 1:** Characteristics of the study group. Mean values ± SD.

	CD (*N* = 35)	UC (*N* = 36)	FGID (control group) **(***N* = 29)
Boys (*N* (%))	26 (74.2%)	21 (58.3%)	8 (27.5%)
Age (years)	15.6 ± 1.9 (range 10.7–18)	13.5 ± 3.7 (range 3.8–18)	13.1 ± 3.9 (range 4.5–17.8)
Duration of disease (months)	33 ± 30	23 ± 27	**—**
BMI (kg/m^2^)	18.9 (17.1–21.8)	19.3 (16.2–20.9)	18.1 (15.9–20.7)
BMI (*Z*-score)	−0.36 (−1.88–0.53)	0.01 (−0.98–0.71)	−0.41 (−82–0.25)

**Table 2:** Total antioxidative status/capacity (TAS/TAC), total oxidative status/capacity (TOS/TOC), and oxidative stress index (OSI) in the study group. Median values and interquartile range (1–3 Q).

	CD (*N* = 35)	CU (*N* = 36)	FGID (control group) (*N* = 29)	Statistical significance (*p* value)
BMI (kg/m^2^)	20.1 (17.1–21.8)	18.9 (16.2–20.9)	18.4 (15.9–20.7)	0.4
IMC (cm)	0.4 (0.34–0.41)	0.43 (0.37–0.48)	0.405 (0.34–0.46)	0.2
TCH (mg/dl)	128.9 (111.2–142.5)	140.2 (116.7–160.7)	160.3 (131–185)	0.007
LDL (mg/dl)	68.2 (45.8–88.2)	73.8 (51.6–92.7)	93.2 (73.5–109.2)	0.009
HDL (mg/dl)	43.6 (−47.7)	49.1 (41–56.9)	50.0 (40.7–57.4)	0.1
TG (mg/dl)	84.9 (59–103)	90.5 (57–109)	85 (55–97)	0.7
Anti-oxLDL (U/ml)	8058 (207–7863)	6118 (242–7475)	4703 (842–7000)	0.7
oxLDL (ng/ml)	213.9 (12.8–167.9)	141.3 (17.5–182.4)	277.6 (15.8–379.1)	0.09
TAS/TAC (*μ*mol/l)	270 (245–288)	262 (236–302)	262 (199–278)	0.4
Low (*N* (%))	22 (62.9%)	23 (63.9%)	23 (79.3%)	0.4
Moderate (*N* (%))	8 (22.8%)	9 (25%)	2 (6.9%)	0.4
High (*N* (%))	5 (14.3%)	4 (11.1%)	4 (13.8%)	0.4
TOS/TOC (*μ*mol/l)	621 (373–1360)	853 (289–1899)	684 (428–1374)	0.9
Low (*N* (%))	5 (14.3%)	8 (22.2%)	2 (6.9%)	0.5
Mild (*N* (%))	3 (8.6%)	2 (5.5%)	2 (6.9%)	0.5
High (*N* (%))	27 (77.1%)	26 (72.3%)	25 (86.2%)	0.5
OSI	244 (131–513)	305 (115–755)	353 (161–534)	0.8

**Table 3:** oxLDL, anti-oxLDL, total antioxidative status/capacity (TAS/TAC), total oxidative status/capacity (TOS/TOC), and oxidative stress index (OSI) according to the severity of disease progression assessed in PCDAI/PUCAI scales in the combined group of children with Crohn's disease and ulcerative colitis. Median values and interquartile range (1–3 Q).

	Severe (*N* = 8)	Moderate (*N* = 17)	Mild (*N* = 22)	Remission (*N* = 21)	Statistical significance (*p* value)
Anti-oxLDL (U/ml)	8200 (71–14,680)	7082 (237–8552)	5341 (258–6349)	8372 (252–8571)	0.93
oxLDL (ng/ml)	349.9 (8.5–130.8)	109.4 (15.6–191.2)	103.6 (12.5–149.3)	251.4 (24.9–348.7)	0.21
TAS/TAC (*μ*mol/l)	276 (259–320)	272 (248–285)	259 (227–286)	262 (232–315)	0.24
TOS/TOC (*μ*mol/l)	659 (474–2060)	1082 (432–2444)	804 (228–2224)	748 (288–1333)	0.78
OSI	260 (154–747)	369 (156–804)	274 (127–889)	245 (125–469)	0.8

## Data Availability

The data used to support the findings of this study are available from the corresponding author upon request.
